# LASIK for Spherical Refractive Myopia: Effect of Topographic Astigmatism (Ocular Residual Astigmatism, ORA) on Refractive Outcome

**DOI:** 10.1371/journal.pone.0124313

**Published:** 2015-04-15

**Authors:** Andreas Frings, Gisbert Richard, Johannes Steinberg, Christos Skevas, Vasyl Druchkiv, Toam Katz, Stephan J. Linke

**Affiliations:** Department of Ophthalmology, University Medical Centre Hamburg-Eppendorf (UKE), Hamburg, Germany; University of Missouri-Columbia, UNITED STATES

## Abstract

**Purpose:**

In eyes with a preoperative plano refractive cylinder, it would appear that there is no rationale for astigmatic treatment. The aim of this retrospective, cross-sectional data analysis was to determine the amount of topographic astigmatism in refractive plano eyes that results in reduced efficacy after myopic laser in situ keratomileusis (LASIK).

**Methods:**

This study included 267 eyes from 267 consecutive myopic patients with a refractive plano cylinder. Receiver operating characteristic analysis was used to find the cut-off values of preoperative ocular residual astigmatism (= topographic astigmatism) that can best discriminate between groups of efficacy and safety indices in preoperative plano refractive cylinder eyes.

**Results:**

Preoperative ocular residual astigmatism (ORA) (or topographic astigmatism) of ≤0.9 diopters (D) resulted in an efficacy index of at least 0.8 statistically significantly more frequently than eyes with a preoperative ORA of >0.9 D. Eyes with a high ORA preoperatively also had a high ORA postoperatively. Regression analysis showed that each diopter of preoperative ORA reduced efficacy by 0.07.

**Conclusion:**

A preoperative corneal astigmatism of ≥0.9 D could (partially) be taken into account in the LASIK design, even if the subjective refractive astigmatism is neutral.

## Introduction

The results of previous studies[1,2–4] have suggested that treating astigmatism with laser in situ keratomileusis (LASIK) based on manifest subjective refraction results in a more successful outcome if the preoperative refractive astigmatism arises primarily from the anterior corneal surface. However, in eyes with a preoperative plano refractive cylinder there is no rationale for astigmatic treatment and, therefore, any postoperative refractive cylinder is either induced by the ablation, the flap preparation or both. Recently, we assessed the influence of specific parameters on pre-existing ocular residual astigmatism (ORA) in patients scheduled for LASIK to treat myopic astigmatism.[5] To determine the amount of ORA before LASIK, we performed a standard double-angle vector analysis in which the magnitude and orientation of ORA were determined by the vector difference between the preoperative refractive astigmatism (R) (corneal plane) and the topographic (simulated keratometry [K]) astigmatism.[1,2–4] Following Kugler et al.[1], the R value was obtained from the manifest refraction, and the simulated K value was calculated from corneal topography based on the difference between the steepest meridian and the flattest meridian oriented at 90 degrees to each other. The ratio of ORA to preoperative refractive cylinder (R) was calculated for each patient. However, in eyes without preoperative refractive cylinder this value cannot be applied.

The aim of the present study was to determine the amount of topographic astigmatism (= the amount of ORA) in refractive plano eyes that results in reduced efficacy after myopic LASIK. The current study was based on the Hamburg Refractive Database and was undertaken to investigate the effect of applying the new definition of high and low ORA for eyes with pre-existing plano refractive cylinder using ROC (receiver operating characteristic) analysis.

## Methods

### Patients and Methods

This retrospective study included 267 eyes from 267 consecutive myopic patients treated between December 2010 and March 2013, and was based on the Hamburg Refractive Database (data retrieved from Care Vision Refractive Centres in Germany). In all eyes a refractive plano cylinder was present preoperatively. Patients with a significant pre-existing complication of the ocular surface or tear film, and eyes with intra- and postoperative flap complications were excluded from analysis. The latter included eyes with postoperatively dislocated flap, epithelial ingrowth, diffuse lamellar keratitis and central toxic keratopathy. Written informed consent for retrospective data analysis was obtained from refractive surgery candidates during their recruiting process. The study and consent procedure were approved by the local ethics committee of the University of Hamburg, Germany (no. 2882), and adhered to the tenets of the Declaration of Helsinki. Manifest spherical and cylindrical refractions, as well as visual acuity with and without correction were assessed preoperatively and 1 day, and 1, 3–4, and 6 months postoperatively, and recorded electronically. All outcome results reported here are based on the data from the 1-month follow-up (Table 1). The spherical and cylindrical refractions were acquired by subjective refraction, and topographic cylinder was obtained using Pentacam Scheimpflug topography (Oculus, Wetzlar, Germany). All refractions were acquired by subjective refraction by expert optometrists in different refractive centres using the same refractometers, visus tables and documentation protocol. Each patient was examined pre- and postoperatively by the same optometrist. Room conditions were standardized according to a protocol written by the medical director of Care Vision Germany (T.K.).The Alpins vector method, which has been described elsewhere [2–4], was applied to describe the effects of LASIK on refractive cylinder. Target induced astigmatic vector (TIA) is the astigmatic change (by magnitude and axis) the surgery is intended to induce. Surgically induced astigmatic vector (SIA) is the amount and axis of astigmatic change the surgery actually induces. It ideally should equal the TIA and can also be described as vector of the real change achieved by surgery.[2–4]

**Table 1:** Descriptives.

Preoperative ORA(1)	Low (<0.9) ORA (n = 153)		High (≥0.9) ORA (n = 114)		Total (N = 267)		P(2)
	Min/Max	Mean (SD)	Min/Max	Mean (SD)	Min/Max	Mean (SD)	
age (y)	19/68	33(±10)	19/63	35(±10)	19/68	34(±10)	0.236
Scotopic pupil size (mm)	4.0/8.0	6.5(±0.7)	4.5/9.0	6.5(±0.7)	4.0/9.0	6.5(±0.7)	0.513
**Preop refractive data**							
CDVA(3) (LogMar)	-0.12/0.15	-0.03(±0.05)	-0.14/0.34	-0.02(±0.06)	-0.14/0.34	-0.02(±0.05)	0.077
UDVA(4) (LogMAr)	0.00/2.00	1.18(±0.67)	0.00/2.00	1.18(±0.62)	0.00/2.00	1.18(±0.65)	0.995
sphere (D)	-8.00/-0.75	-3.58(±1.49)	-8.00/-1.00	-3.74(±1.77)	-8.00/-0.75	-3.65(±1.62)	0.413
topographic cyl (D) (= ORA magnitude (D))	-0.80/-0.10	-0.50(±0.21)	-2.00/-0.90	-1.11(±0.22)	-2.00/-0.10	-0.76(±0.37)	0.000
ORA axis (°)	5/174	88(±31)	21/178	89(±14)	5/178	89(±25)	0.796
**Postop refractive data**							
CDVA (LogMar)	-0.20/0.10	-0.04(±0.05)	-0.18/0.19	-0.02(±0.07)	-0.20/0.19	-0.03(±0.06)	0.010
UDVA (LogMAr)	-0.20/0.44	-0.01(±0.08)	-0.16/2.00	0.05(±0.22)	-0.20/2.00	0.01(±0.16)	0.001
sphere (D)	-0.75/2.50	0.24(±0.46)	-1.75/2.50	0.22(±0.56)	-1.75/2.50	0.23(±0.51)	0.793
subjective cyl (D)	-1.25/-0.25	-0.40(±0.19)	-2.00/-0.25	-0.47(±0.33)	-2.00/-0.25	-0.43(±0.26)	0.030
topographic cyl (D)	-1.90/0.00	-0.65(±0.37)	-2.10/-0.20	-1.13(±0.38)	-2.10/0.00	-0.86(±0.44)	0.000
Spherical Equivalent (D)	-1.00/2.13	0.04(±0.43)	-2.00/2.13	-0.01(±0.56)	-2.00/2.13	0.02(±0.49)	0.400
ORA magnitude (D)	0.05/1.54	0.55(±0.31)	0.05/2.46	0.95(±0.42)	0.05/2.46	0.72(±0.41)	0.000
ORA axis (°)	3/175	91(±34)	9/152	93(±20)	3/175	92(±29)	0.606
Refractive SIA(5) magnitude (D)	0.25/1.25	0.40(±0.19)	0.25/2.00	0.47(±0.33)	0.25/2.00	0.43(±0.26)	0.030
Refractive SIA (°)	4/180	83(±46)	5/180	81(±36)	4/180	83(±42)	0.721
Refractive TSIA(6) magnitude (D)	0.03/1.73	0.43(±0.30)	0.00/3.09	0.50(±0.45)	0.00/3.09	0.46(±0.37)	0.111
Refractive TSIA (°)	1/180	86(±47)	0/177	93(±55)	0/180	89(±51)	0.273
Efficacy Index	0.36/1.33	0.98(±0.15)	0.01/1.25	0.91(±0.21)	0.01/1.33	0.95(±0.18)	0.002
Safety Index	0.70/1.39	1.04(±0.12)	0.64/1.39	1.02(±0.12)	0.64/1.39	1.03(±0.12)	0.255

1 = Ocular residual astigmatism; 2 = P value <0.05 was considered as significant; 3 = corrected distance visual acuity; 4 = uncorrected distance visual acuity; 5 = refractive surgically induced astigmatism (subjectively manifest SIA); 6 = topographic SIA; Means of astigmatism were calculated by arithmetic means.

### Surgical Treatment

The LASIK procedure included mechanical flap preparation using automated microkeratomes (MKs) from Moria, France. Each MK uses single-use heads with a pre-defined distance of 90μm between the footplate and the oscillating blade. All have one oscillating motor and a second forward /backward advancing motor operated by the surgeon with foot pedals. The MKs are attached to a pump-driven vacuum ring fixed to the limbus. The ring size and blade-progression-stop point were chosen by the surgeon according to the corneal keratometry, the desired flap diameter and hinge-width recommended by the manufacturer. Both eyes of the same patient were operated using the same MK and the same head.

Excimer ablation for all eyes was performed using an Allegretto excimer laser platform (Eye-Q 200 Hertz (Hz) or 400 Hz, WaveLight GmbH, Erlangen, Germany) under constant eye tracking (250 Hz). To minimize the induced spherical high-order aberration (HOA), an aspherical “wavefront-optimized” profile was used with an optical zone of 6.0, 6.5 or 7.0 mm depending on the mesopic pupil diameter and expected residual stromal bed.[6] The manufacturer-recommended “WaveLight myopic astigmatic nomogram” was implemented to compensate for very short or long ablation time and for a cylinder-sphere coupling effect. However, cylinder magnitudes of 0.25 D or less are not addressed by this nomogram. Cyclotorsion was minimized using a “NeuroTrack” system (WaveLight GmbH) in which four built-in blinking light sources eliminate cyclotorsion at its source by controlling optokinesis.

The laser treatments were performed in eight Refractive Centres located in Berlin, Cologne, Frankfurt/ Main, Hamburg, Hanover, Munich, Nuremberg, and Stuttgart. All refractive surgeons were senior consultants who had performed at least 500 LASIK surgeries and who followed a standard protocol of indications, and preoperative, intraoperative and postoperative management written by the first author (T.K.); T.K. also trained the consultants at their own centres. Previously, to rule out systematic differences between 200- and 400-Hz lasers, and thus between our centres, a Kruskal–Wallis test was applied and demonstrated that there were no systematic differences.[7] Postoperative preservative-free medication after LASIK included ofloxacin four times a day for 1 week, and dexamethasone four times a day for the 1st week, and two times a day for the 2nd and 3rd weeks. Hyaluronic acid artificial tears (Hylolasop, Ursapharm GmbH, Germany) were applied to all eyes for 1–4 months.

### Statistical analysis

ROC analysis was used to find the cut-off values of preoperative ORA (topographic astigmatism) that can best discriminate between the groups of efficacy (EI) and safety (SI) indices in preoperative plano refractive cylinder eyes. For EI, these groups were ≤0.7 vs. >0.7; ≤0.8 vs. >0.8; ≤0.9 vs. >0.9 and ≤1.0 vs. >1.0. For SI, these groups were ≤0.9 vs. >0.9; ≤1.0 vs >1.0; ≤1.1 vs >1.1 and ≤1.2 vs 1.2. EI was defined as the mean of ratio of postoperative UDVA to preoperative CDVA; SI was defined as the mean of ratio of postoperative CDVA to preoperative CDVA.

The area under the curve (AUC), sensitivity and specificity for the given cut-off values are shown in Table 2. The hypothesis that the AUC is significantly different from 0.5 was tested and the P values were reported. A P value <0.05 was considered as significant. Differences in preoperative and postoperative parameters between the groups of high and low ORA (determined by ROC analysis) were tested using the t-test for independent samples. The differences in nominal scaled parameters such as sex (male, female), eye dominance (dominant, non-dominant), and eye side (left, right) were tested using the Chi-squared test. We also performed an ordinary least square regression (OLS) to estimate the direction and degree of correlation between preoperative ORA magnitude and EI or SI of the operative outcome.

**Table 2:** ROC (receiver operating characteristic) analysis.

Cut-off	ORA(1) cut-off	AUC(2)	SE(3)	Sensitivity	Specificity	P(4)
**EI(5)**						
0.7	0.900	0.354	0.061	0.397	0.280	0.016
0.8	0.900	0.376	0.049	0.382	0.362	0.013
0.9	0.800	0.407	0.039	0.473	0.434	0.017
1.0	0.800	0.456	0.039	0.480	0.490	0.260
**SI(6)**						
0.9	0.8	0.511	0.068	0.508	0.552	0.872
1	0.8	0.463	0.036	0.496	0.493	0.304
1.1	0.8	0.509	0.045	0.537	0.507	0.847
1.2	0.8	0.493	0.061	0.583	0.506	0.915

1 = ocular residual astigmatism; 2 = Area under the curve; 3 = Spherical Equivalent; 4 = P value <0.05 was considered as significant; 5 = Efficacy Index; 6 = Safety Index.

## Results

### ROC analysis

The ROC analysis (Table 2) shows that eyes with a preoperative ORA (or topographic astigmatism) of ≤0.9 diopters (D) reached an EI of at least 0.8 (best sensitivity and high specificity) statistically significantly more frequently than eyes with a preoperative ORA of >0.9 D. If the preoperative ORA or topographic astigmatism was maximally 0.8 D, then an EI of at least 0.9 (statistically significant) or >0.9 (probable) was possible, although with a lower sensitivity and specificity. Therefore, an ORA cut-off of 0.9 was chosen for the comparison of the high and low preoperative ORA or topographic cylinder groups (Table 1). For an SI of 0.9 or more, the preoperative ORA (topographic cylinder) should be maximally 0.8. Sex, eye dominance and side were equally distributed among the groups (Table 1).

### Crosstabulation

The crosstabulation (Table 3) shows that smaller values of preoperative ORA (= topographic astigmatism) resulted in a statistically significantly better EI (Chi-Square = 10.41, P = 0.001). Table 3 also shows that in 62% of the observed cases higher ORA values (≥0.9) were correlated with a low EI (true positive).

**Table 3:** Ordinary least square regression (OLS) analysis.

		True class		
		Low (EI(1) 0.01–0.8)	High (EI 0.82–1.33)	Total
**Predicted class**	**Low (ORA(2) 0.1–0.9)**	17	136	153
	**High (ORA 0.91–2)**	30	84	114
	**Total**	47	220	267
	*True positive*	0.38		
	*False positive rate*	0.64		
	*Sensitivity*	0.38		
	*Specificity*	0.36		
	*Correctly classified*	0.38		

1 = Efficacy Index; 2 = ocular residual astigmatism

### Refractive results I: Sphere, ORA and Visual acuity

No statistically significant difference was found in the preoperative sphere (Table 1). On average, ORA was 0.51±0.21 D (range 0.1–0.8 D) in the low ORA group and 1.11±0.22 D (range 0.9–2.00 D) in the high ORA group; this difference was statistically significant (P<0.001).

There were also no statistically significant differences in the postoperative manifest sphere, but significant differences were found for the postoperative corrected (CDVA) and uncorrected (UDVA) distance visual acuity for eyes with low preoperative ORA (Table 1).

### Refractive results II: subjectively manifest SIA and topographic SIA

The difference in the postoperative subjective cylinder or refractive surgically induced astigmatism (RSIA, subjectively manifest SIA) was statistically significant (P = 0.03), since the eyes of the group with preoperatively higher ORA had, on average, a higher postoperative subjective cylinder (RSIA) with a wider range of results. Therefore, eyes with a high ORA preoperatively also had a high ORA postoperatively; the difference in post-op ORA magnitude between the low and high ORA groupswas statistically significant (P<0.001). The difference in the postoperative topographic astigmatism was also significant (P<0.001) since the eyes of the group with a high ORA preoperatively had, on average, a higher postoperative topographic cylinder.

The RSIA mostly corresponded in value and alignment to the postoperative topographic SIA (TSIA). Eyes with a preoperatively higher ORA had a higher RSIA and TSIA postoperatively, which was statistically significant for RSIA (P = 0.030).

### Refractive efficacy

The differences between CDVA and UDVA were significant for the group with low preoperative ORA.

Therefore, the differences in ORA after LASIK were also significant (P<0.001). In addition, the difference in EI was thus statistically significant (P = 0.002) since here the average value was higher. Significantly higher EI was reached in eyes with a low preoperative ORA.

The bivariate OLS regression (Table 4) shows that there was a statistically significant negative correlation between preoperative ORA magnitude and efficiency index. Each diopter of preoperative ORA reduced efficiency by 0.07. The correlation between preoperative ORA and safety index was not significant.

**Table 4:** Bivariate ordinary least square regression (OLS) analysis.

	Coefficient	Std. Err.	t(2)	P(3)	95% Conf. Interval	
**EI(1)**						
**ORA(4) mangnitude**	-0.07	0.03	-2.37	0.02	-0.13	-0.01
**_cons**	1.00	0.03	39.73	0.00	0.95	1.05
**SI(5)**						
**ORA mangnitude**	-0.02	0.02	-0.80	0.43	-0.05	0.02
**_cons**	1.04	0.02	62.22	0.00	1.01	1.07

1 = Efficacy Index; 2 = empirical t value (coefficient/SE); 3 = significance; 4 = ocular residual astigmatism; 5 = Safety Index

## Discussion

The contributors to refractive astigmatism are the anterior cornea and ORA. The ORA mainly results from the posterior corneal surface, the crystalline lens and some unknown “retinal” components.[8] ORA is defined as the vectorial difference between the corneal topographic astigmatism and the refractive cylinder at the corneal plane.[1,9,10] Our results show that a low preoperative topographic astigmatism or low ORA was correlated with a low postoperative ORA and better refractive results (EI, SI, CDVA). These differences were statistically significant. Therefore, our findings indicate that caution is recommended when a preoperative corneal astigmatism of ≥0.9 D is present. This could favourably be taken into account in the LASIK design, even if the subjective refractive astigmatism is neutral. We further hypothesize that in order to analyze the effect on the postoperative refractive cylinder in such cases, initially, in small trial and error steps maybe a part of the preoperative corneal topographic astigmatism should be corrected. This goal can favorably be reached by applying vector analysis according to Alpins.[2,10,11] Further prospective studies are needed to analyze this assumption and its real effect on post-LASIK refractive results.

In a previous study, we studied 2991 eyes from 2991 consecutive myopic patients scheduled to undergo LASIK to investigate the influence of age, gender, ocular dominance, subjective cylinder and topographic astigmatism, subjective sphere, and mesopic pupil size on pre-existing ORA.[3] The ORA was determined using Alpins vector analysis. Patients were assigned to 1 of 2 subgroups defined by the ratio of ORA to preoperative refractive cylinder (R) (ORA/R of ≥1.0 vs. <1.0). Our analysis indicated that the preoperative assessment of refractive surgery candidates should consider the interaction between topographic, refractive and ocular residual astigmatism. The better the correlation between the magnitude and the orientation of the corneal astigmatism and refractive cylinder, the less astigmatism will remain in the optical system of the eye after treatment.[8,12]

However, the definition of high or low ORA cannot be applied in eyes without preoperative refractive cylinder. Therefore, ROC analysis was used to find cut-off values of preoperative ORA that can best discriminate between the groups of EI and SI.

The ROC analysis (Table 2) shows that eyes with a preoperative ORA or preoperative topographic cylinder of ≤0.9 D attained an EI of at least 0.8 (i.e. best sensitivity and high specificity) statistically significantly more frequently than eyes with a preoperative ORA or topographic astigmatism of >0.9. The difference in postoperative subjective cylinder or RSIA was statistically significant (P = 0.03) since the eyes of the patients in the group with a high ORA preoperatively on average presented a higher postoperative subjective cylinder or RSIA with a wider range of results. The difference in EI was therefore also statistically significant (P = 0.002). A statistically higher EI was also obtained for eyes with a low ORA preoperatively.

Both corneal and non-corneal astigmatism are usually balanced. In a recent study, the amount of aberration of both the cornea and internal optics was found to be larger than that for the complete eye, indicating that the first surface of the cornea and internal optics partially compensate for each other’s aberrations and produce an improved retinal image.[13] As a consequence, topographic astigmatism and refractive astigmatism do not necessarily coincide in magnitude and axis.[2–4,10,11] Moreover, on average, 40% less of the ORA being corrected on the cornea would reduce the corneal astigmatism significantly without compromising the refractive astigmatism outcome.[2–4,11] According to Alpins[2–4], using vector planning that integrates topographic values can more effectively reduce overall remaining astigmatism. Integrating the topography parameters with the wavefront aberrometry results in greater reduction in corneal astigmatism and better visual outcomes under mesopic conditions.[11] The initial study of Alpins et al.[2] of 100 patients found a mean ORA value of 0.81 D; 34% of patients had a value greater than 1.00 D.

The strength of our study includes a large sample size, homogeneity of the method of measurement of refraction, and a strict exclusion of ocular pathologies. On the other hand, we have to admit that statistically significant results do not necessarily indicate clinically relevant differences. There are other factors besides those described in our study that affect the astigmatic status and ORA. These are the posterior cornea, vitreous, and retina, as well as non-optical components such as the visual cortex.[1,14] Of course, we also cannot totally exclude measurement errors in manifest cylinder, although this was addressed by a standard protocol. Accordingly, our conclusion should be carefully qualified as being determined in a sample of patients with spherical myopia by refraction but showing some anterior corneal astigmatism on topography. By definition, in an eye where the refractive astigmatism was underestimated in the manifest refraction, this eye will be classified as having high ORA. Postoperatively, if the astigmatism is then picked up on the manifest refraction, this will be interpreted as having been induced by the procedure, however it seems more likely that there was an error in the preoperative manifest refraction—at least in some cases. Certainly, in our clinic, we have observed this where a patient has been refracted with no astigmatism preoperatively but has topographic astigmatism and astigmatism on autorefraction, who then has astigmatism in the manifest refraction postoperatively.

## Conclusion

Our results show that a refractive, purely spherical, myopia and preoperatively low topographic astigmatism or low ORA correlated with a low postoperative ORA and better refractive results after LASIK. The differences were statistically significant. Therefore, our data indicate that a preoperative corneal astigmatism of ≥0.9 D could (partially) be treated at the same time even when the subjective refractive astigmatism is neutral. This goal can favorably be reached by applying vector analysis according to Alpins.[2,8,9] We suggest that, in such cases, 50% of the preoperative corneal topographic astigmatism should be corrected initially to analyze the effect on the postoperative refractive cylinder.
